# Positive Modulation of Angiotensin II Type 1 Receptor–Mediated Signaling by LVV–Hemorphin-7

**DOI:** 10.3389/fphar.2019.01258

**Published:** 2019-10-25

**Authors:** Amanat Ali, Abdulrasheed Palakkott, Arshida Ashraf, Isra Al Zamel, Bincy Baby, Ranjit Vijayan, Mohammed Akli Ayoub

**Affiliations:** Department of Biology, College of Science, United Arab Emirates University, Al Ain, United Arab Emirates

**Keywords:** hemorphin, AngII, AT1R, GPCR, BRET, RAS, allosteric modulation, hypertension

## Abstract

Hemorphins are hemoglobin β-chain–derived peptides initially known for their analgesic effects *via* binding to the opioid receptors belonging to the family of G protein–coupled receptor (GPCR), as well as their physiological action on blood pressure. However, their molecular mechanisms in the regulation of blood pressure are not fully understood. Studies have reported an antihypertensive action *via* the inhibition of the angiotensin-converting enzyme, a key enzyme in the renin–angiotensin system. In this study, we hypothesized that hemorphins may also target angiotensin II (AngII) type 1 receptor (AT1R) as a key GPCR in the renin–angiotensin system. To investigate this, we examined the effects of LVV–hemorphin-7 on AT1R transiently expressed in human embryonic kidney (HEK293) cells using bioluminescence resonance energy transfer (BRET) technology for the assessment of AT1R/Gαq coupling and β-arrestin 2 recruitment. Interestingly, while LVV–hemorphin-7 alone had no significant effect on BRET signals between AT1R and Gαq or β-arrestin 2, it nicely potentiated AngII-induced BRET signals and significantly increased AngII potency. The BRET data were also correlated with AT1R downstream signaling with LVV–hemorphin-7 potentiating the canonical AngII-mediated Gq-dependent inositol phosphate pathway as well as the activation of the extracellular signal–regulated kinases (ERK1/2). Both AngII and LVV–hemorphin-7–mediated responses were fully abolished by AT1R antagonist demonstrating the targeting of the active conformation of AT1R. Our data report for the first time the targeting and the positive modulation of AT1R signaling by hemorphins, which may explain their role in the physiology and pathophysiology of both vascular and renal systems. This finding further consolidates the pharmacological targeting of GPCRs by hemorphins as previously shown for the opioid receptors in analgesia opening a new era for investigating the role of hemorphins in physiology and pathophysiology *via* the targeting of GPCR pharmacology and signaling.

## Introduction

Hemorphins are endogenous hemoglobin-derived peptides that were initially reported to have opiate-like activity. They are normally released from hemoglobin beta-chain during physiological or pathophysiological conditions. These peptides are 4 to 10 amino acids long and share a common Tyr-Pro-Trp-Thr tetrapeptide core (YPWT or hemorphin-4) (Liebmann et al., 1989; Zhao et al., 1995; Ivanov et al., 1997; Nyberg et al., 1997). A number of different hemorphin peptides have been identified including hemorphin-4, hemorphin-7, and LVV–hemorphin-4, -6, and -7 (Brantl et al., 1986; Piot et al., 1992; Dagouassat et al., 1996; Moisan et al., 1998), which are naturally produced in the brain, spinal cord, cerebrospinal fluid, and plasma (Erchegyi et al., 1992; Glämsta etal., 1992; Karelin et al., 1994). Among hemorphin peptides, LVV–hemorphin-7 (LVVYPWTQRF) is the longest and the most stable form and possesses the highest hydrophobicity. It is also the most abundant hemorphin in the mammalian central nervous system (Karelin et al., 1994; Moeller et al., 1997).

Hemorphins have been shown to possess a number of biological activities including effects on spatial learning, inflammation, analgesia, and transient hypotension (Hughes et al., 1975; Moisan et al., 1998; Sanderson et al., 1998; Albiston et al., 2004; Cejka et al., 2004; Lee et al., 2004; Cheng et al., 2012) The analgesic effects of hemorphins have been attributed to its pharmacological action on the opioid receptors belonging to the G protein–coupled receptor (GPCR) family (Davis et al., 1989; Liebmann et al., 1989; Glämsta et al., 1992; Yukhananov et al., 1994; Szikra et al., 2001; Chow et al., 2018). LVV–hemorphin-7 has also been reported to bind and inhibit the angiotensin IV (AngIV) receptor, or the insulin-regulated aminopeptidase, in the hippocampus with significant improvement of memory (Albiston et al., 2004; Lee et al., 2004). *In vitro* and *in vivo* studies have demonstrated the beneficial effects of hemorphins in the control of blood pressure. In spontaneously hypertensive rats, a significant decrease in blood pressure and heart rate was observed after an intraperitoneal injection of LVV–hemorphin-7 (Cejka et al., 2004). Furthermore, in anesthetized rats, hemorphins have been shown to increase the hypotensive effect of bradykinin (Ianzer et al., 2006). *In vitro* studies have demonstrated that hemorphins inhibit angiotensin-converting enzyme (ACE), a key enzyme in the renin–angiotensin system (RAS) (Lantz et al., 1991; Zhao and Piot, 1997; Fruitier-Arnaudin et al., 2002). In fact, ACE is the key RAS component that leads to the release of the vasoconstrictor peptide AngII. AngII mediates its physiological functions by binding to specific GPCRs–AngII type 1 receptor (AT1R) and AngII type 2 receptor (Johnston, 1992; Inagami, 1999; Hunyady and Catt, 2006). At the molecular level, AngII-induced AT1R activation results in its coupling to Gαq/11 proteins triggering inositol triphosphate production and Ca^2+^ release (Johnston, 1992; Inagami, 1999; Hunyady and Catt, 2006). AT1R also signals through other G protein–independent signaling pathways, such as β-arrestin–mediated extracellular signal–regulated kinase (ERK1/2) activation and other pathways (Johnston, 1992; Inagami, 1999; Hunyady and Catt, 2006).

In this study, we attempted to link the role of hemorphins in the regulation of blood pressure and RAS with its putative direct action on AngII receptors. We hypothesized that in addition to their action on ACE hemorphins may also pharmacologically target AT1R as the key GPCR in RAS. To test this, we examined the effects of LVV–hemorphin-7 on the activation of AT1R transiently expressed in HEK293 using bioluminescence resonance energy transfer (BRET) technology, which allows the real-time assessment of the functional AT1R/Gαq coupling as well as β-arrestin 2 recruitment in live cells. Moreover, we examined the functional effect of LVV–hemorphin-7 on AT1R-mediated downstream signaling pathways by measuring the cytoplasmic Gαq-dependent IP1 production and ERK1/2 phosphorylation. 

## Methods

### cDNA Constructs and Ligands

The following human protein-coding plasmids were used for the transient expression in HEK293 cells: AT1R-RlucII, yPET–β-arrestin 2, and Venus-Gαq were generously provided by Dr. Stéphane Laporte (McGill University, Montréal, QC, Canada), Dr. Mark Scott (Cochin Institute, Paris, France), and Dr. Nevin Lambert (Augusta University, GA, USA), respectively. AngII and irbesartan (Sigma, St. Louis, MO, USA) and LVV–hemorphin-7 (LVVYPWTQRF) (New England Peptide, Gardner, MA, USA) were used as ligands.

### Cell Culture and Transfection

HEK293 cells were maintained at 37°C, 5% CO_2_ in complete medium (Dulbecco modified Eagle medium (DMEM) containing 0.3 mg/ml glutamine, 100 IU/ml penicillin, and 100 µg/ml streptomycin) supplemented with 10% fetal calf serum (GIBCO BRL, Carlsbad, CA, USA). Transient transfections for BRET were carried out in 96-well plates and IP1 and ERK1/2 assays in six-well plates using Lipofectamine 2000 (Invitrogen). Briefly, for BRET assays in each 96-well plate, 25 ng of AT1R-Rluc was mixed with 50 ng of either Venus-Gαq or yPET–β-arrestin 2 in 25 µl of serum-free DMEM and incubated for 5 min at room temperature. The plasmid solution was then mixed with 25 µl of serum-free DMEM containing 0.5 µl of Lipofectamine 2000 and incubated for 20 min at room temperature. Cells (10^5^ in 150 µl/well) resuspended in DMEM supplemented with 10% fetal calf serum (FCS) were then incubated with the final DNA-Lipofectamine 2000 mix (50 µl/well) and seeded in 96-well plates. All assays were carried out 48 h posttransfection.

### Dose–Response BRET Assay

BRET technology was used for dose–response analysis as previously described (Ayoub et al., 2015; Ayoub, 2016; Ayoub et al., 2016). Cells were first washed with 50 µl/well of phosphate-buffered saline (PBS) and treated for 30 min at 37°C with 40 µL of PBS containing or not (vehicle) the indicated doses of either AngII (control), LVV–hemorphin-7, or AngII in the presence of the indicated doses of LVV–hemorphin-7 (50 nM, 0.5 µM, 10 µM, or 100 µM). Following treatment, 10 µl of coelenterazine h (Promega) in PBS was added to a final concentration of 5 µM, and BRET measurements were carried out using the Tristar 2 multilabel plate reader (Berthold, Germany) allowing sequential measurements of light emission at 480 and 540 nm. For the antagonist experiments with irbesartan, cells were first pretreated with 30 µl of irbesartan (10 µM) for 15 min at 37°C before their treatment with 10 µl of LVV–hemorphin-7 (10 µM) followed by the stimulation with 10 µl of AngII (10 nM) for 30 min at 37°C, and BRET measurements were performed as described above.

### Real-Time BRET Kinetic Assay

For the real-time BRET kinetics, different protocols were used. Cells were first washed with 50 µl/well of PBS and resuspended in 40 µl/well of PBS containing coelenterazine h (5 µM), and BRET signals were measured in real time for ∼5 min to determine the baseline. In the direct protocol, after the baseline, 10 µl/well of PBS containing or not (vehicle) either 10 nM of AngII (control), 10 µM of LVV–hemorphin-7, or both, was added, and BRET measurements were carried out for 50 min (**Figures 3A, B**). In the sequential protocol, after the baseline, BRET signals were measured in real time upon two sequential treatments (T1 and then T2) as indicated in **Figures 3C, D**. This consists of 10 µl/well of PBS containing or not (vehicle) 10 µM of LVV–hemorphin-7 (T1) for 10 min of BRET measurements followed by 10 µl/well of PBS containing or not (vehicle) 10 nM of AngII for 25 min of BRET measurements. The opposite order (10 nM of AngII followed by 10 µM of LVV–hemorphin-7) was also performed as described in **Figures 3E, F**.

### IP1 Accumulation Assay

Measurement of IP1 accumulation was performed in HEK293 cells expressing either AT1R-Rluc with Venus-Gαq like for BRET assays or untagged AT1R (AT1R-WT) alone using the IP-One Tb kit (Cisbio Bioassays, France) according to manufacturer’s instructions. For this, cells were first pretreated or not (control) with 10 µM of LVV–hemorphin-7 for 15 min at 37°C before stimulation or not (vehicle) with the increasing doses of AngII for 30 min at 37°C. The cells were then lysed by adding the supplied assay reagents, and the assay was incubated for 1 h at room temperature. Fluorescence emission was measured at 620 and 665 nm, 50 µs after excitation at 340 nm using the Tristar 2 multilabel plate reader (Berthold, Germany).

### ERK1/2 Phosphorylation

HEK293 cells expressing either AT1R-Rluc with Venus-Gαq like for BRET assays or AT1R-WT and seeded onto a six-well plate at 10^6^ cells/well were first serum starved overnight and then stimulated or not with 10 nM of AngII, 10 µM of LVV–hemorphin-7, or both, at 5 or 15 min at 37°C as indicated in **Figure 4B**. After treatment, cells were washed in ice-cold PBS and lysed with 250 µL/well of ice-cold RIPA buffer (Pierce) containing protease inhibitor cocktail (Sigma) and phosphatase inhibitor tablet (Roche). Cells were then gently lysed for 1 h at 4°C, and the cell lysates were scraped from the wells, transferred into Eppendorf tubes, and centrifuged at 15,000*g* for 15 min at 4°C. The protein concentration in the lysate supernatant was determined using a Pierce BCA Protein Assay Kit (Thermo Fisher Scientific). An equal protein amount from each sample was mixed with Laemmli buffer (BioRad) containing 8% β-mercaptoethanol and heated at 95°C for 5 min for 10% sodium dodecyl sulfate polyacrylamide gel electrophoresis (SDS-PAGE) at 100 V for approximately 1 h. Proteins were then transferred onto polyvinylidene fluoride membrane (BioRad). Membranes were incubated with blocking buffer [5% skimmed milk in PBS containing 0.1% Tween 20 (PBST)] for 1 h at room temperature and washed with PBST. Then, membranes were incubated overnight at 4°C with either primary mouse monoclonal anti–phospho-p44/42 (pERK1/2) (Cell Signaling) (1:2,000 dilution in TBST containing 5% skimmed milk) for the phosphorylated proteins or primary rabbit polyclonal anti-p44/42 ERK1/2 (1:1,000 dilution in TBST containing 5% bovine serum albumin) for total proteins. Membranes were then washed with PBST three times for 5 min with gentle shaking. Horseradish peroxidase–conjugated anti–immunoglobulin G was used as a secondary antibody. After further washing, immunoreactive bands were detected by ECL chemiluminescent substrate (Thermo Fisher Scientific), and chemiluminescence was detected using the LiCOR C-DiGit blot scanner. Densitometry analysis of membrane was performed using Image Studio version 5.2 software.

### Data Presentation and Statistical Analysis

The BRET data given as the ratio of light emission at 540 nm over 480 nm were first converted to “ligand-induced BRET” signals by subtracting the ratio obtained from vehicle-treated cells from the same ratio obtained from AngII/LVV–hemorphin-7–treated cells. Then, the % of responses in BRET and IP1 measurements were obtained by taking as 100% the maximal AngII-induced signal in the control condition in the different assays. All kinetic and the sigmoidal dose–response curves were fitted to appropriate nonlinear regression equations using GraphPad Prism software (San Diego, CA, USA). Statistical analyses were performed with two-way ANOVA and Tukey multiple-comparisons test to determine statistical significance between the different conditions.

## Results

### LVV–Hemorphin-7 Has Weak Agonistic Effects on AT1R

**Figure 1:**
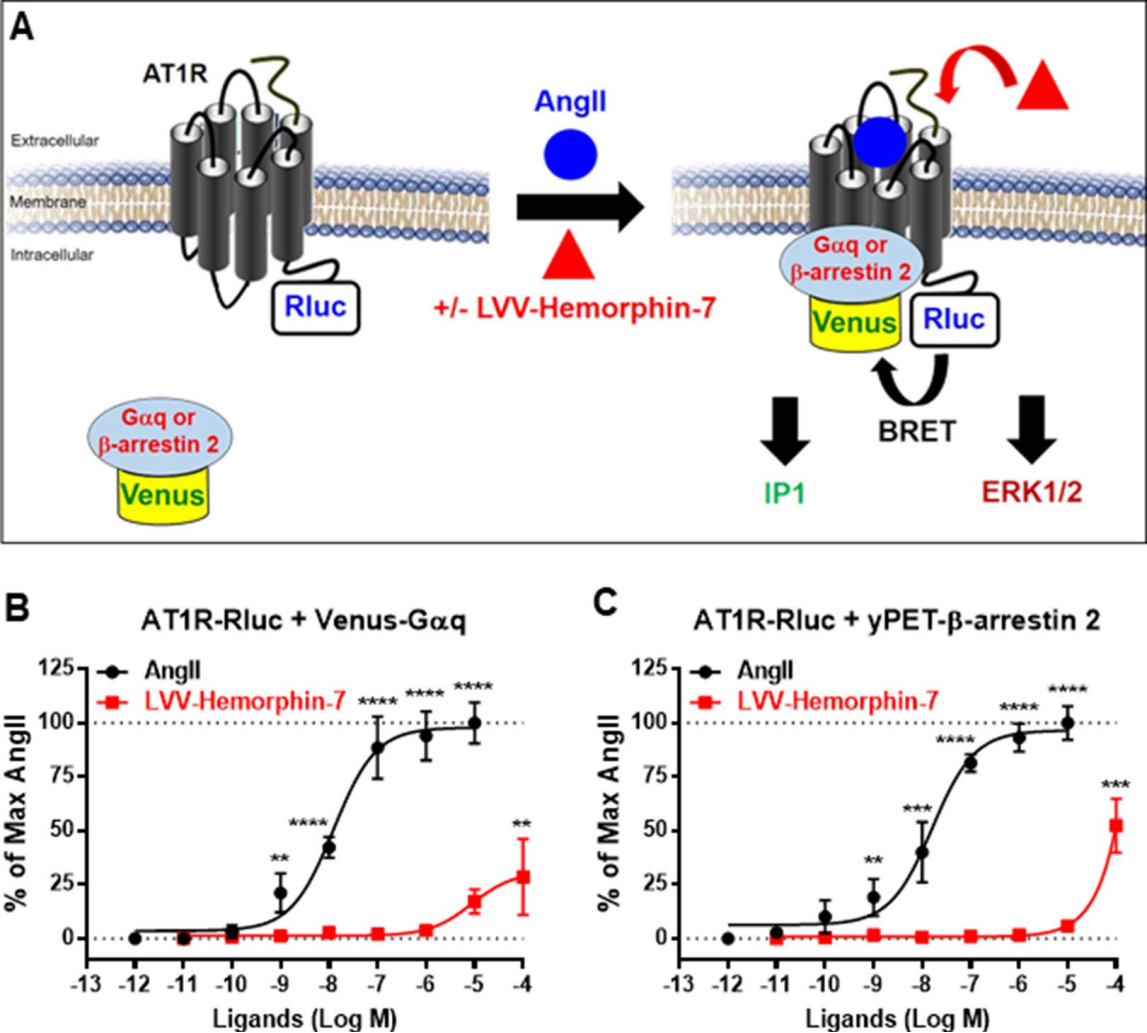
Pharmacological action of LVV–hemorphin-7 on AT1R. **(A)** BRET, IP1, and ERK1/2 assays were used in HEK293 cells transiently coexpressing AT1R-Rluc (as BRET donor) and either Venus-Gαq or yPET–β-arrestin 2 (as BRET acceptors) treated or not with either AngII, LVV–hemorphin-7, or both. For BRET, cells coexpressing AT1R-Rluc and either Venus-Gαq **(B** and **D)** or yPET–β-arrestin 2 **(C** and **D)** were treated with increasing doses of AngII or LVV–hemorphin-7 for 30 min at 37°C and BRET signals were measured. Data are means ± SEM of five independent experiments performed in triplicate. ****p < 0.0001, ***p < 0.001, **p < 0.01, and not statistically significant, *p > 0.05*.

In order to investigate the pharmacological targeting of AT1R with LVV–hemorphin-7 *in vitro*, we used BRET technology in HEK293 cells for the functional AT1R-Gαq coupling and β-arrestin 2 recruitment to AT1R, in real time and live cells, as previously described (Ayoub et al., 2015; Ayoub, 2016; Ayoub et al., 2016) and illustrated in **Figure 1A**. We also investigated Gαq-mediated IP1 production and ERK1/2 activation, as two major AT1R downstream signaling pathways. For BRET assays, we used AT1R-Rluc, as BRET donor, coexpressed with either the mini Gαq (mGsq) probe consisting of the GTPase domain of Gαq fused to Venus, as BRET acceptor, as recently described (Wan et al., 2018), or yPET–β-arrestin 2, in HEK293 cells. BRET measurements were performed upon treatment or not of cells with increasing doses of AngII or LVV–hemorphin-7. This showed that AngII increased the BRET signals between AT1R-Rluc and both Venus-Gαq (**Figure 1B**) and yPET–β-arrestin 2 (**Figure 1C**) in a dose-dependent manner and with the expected potency (**Table 1**). However, only high doses of LVV–hemorphin-7 (10–100 µM) showed a partial effect on AT1R-Gαq coupling (**Figure 1B**) and β-arrestin 2 recruitment (**Figure 1C**). This indicates no strong pharmacological effects of LVV–hemorphin-7 on AT1R at reasonable doses.

**Table 1:** Log EC_50_ of AngII and LVV–hemorphin-7 in the different assays.

Transfections	Log EC_50_ AngII *(BRET)*		Log EC_50_ AngII *(IP1)*		Log EC_50_ LVV–hemorphin-7 *(BRET)*
AT1R-Rluc	Control	LVV–hemorphin-7	Control	LVV–hemorphin-7	−5.41 ± 0.29
+	−7.68 ± 0.17	−8.78 ± 0.14	−7.57 ± 0.19	−8.93 ± 0.17	(n = 5)
Venus-Gαq	(*p* < 0.001, n = 6)		(*p* < 0.0001, n = 7)		
AT1R-Rluc	Control	LVV–hemorphin-7	ND		−5.48 ± 0.35
+	−7.75 ± 0.23	−8.91 ± 0.19			(n = 5)
yPET–β-arrestin 2	(*p* < 0.01, n = 5)				
AT1R-WT	ND		Control	LVV–hemorphin-7	ND
			−8.15 ± 0.03	−9.55 ± 0.03	
			(*p* < 0.0001, n = 3)		

The statistical analysis is indicative of significance compared to the conditions with AngII in the absence of 10 µM of LVV–hemorphin-7 (control).

### Positive Effects of LVV–Hemorphin-7 on AT1R Activation Revealed by BRET

**Figure 2:**
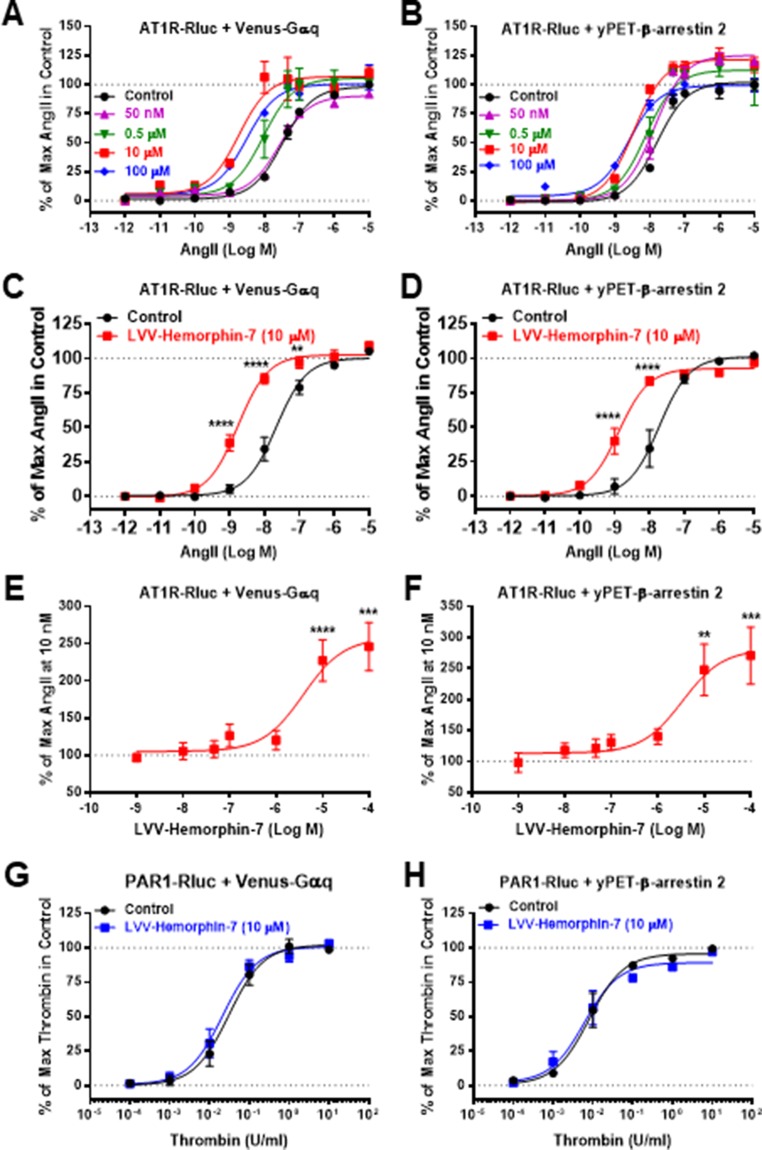
Positive effect of LVV–hemorphin-7 on AT1R activation revealed by dose–response BRET analysis. HEK293 cells transiently coexpressing AT1R-Rluc and either Venus-Gαq **(A**, **C**, and **E)** or yPET–β-arrestin 2 **(B**, **D**, and **F)** were used for dose–response BRET analysis as indicated in *Methods*. In **(A** and **B)**, cells were first pretreated or not (control) for 15 min with different doses of LVV–hemorphin-7 and then stimulated 30 min with increasing doses of AngII. In **(C** and **D)**, cells were first pretreated or not (control) 15 min with 10 µM of LVV–hemorphin-7 and then stimulated 30 min with increasing doses of AngII. However, in **(E** and **F)**, cells were first pretreated for 15 min with increasing doses of LVV–hemorphin-7 and then stimulated 30 min with 10 nM of AngII. As a negative control, cells transiently coexpressing PAR1-Rluc and either Venus-Gαq **(G)** or yPET–β-arrestin 2 **(H)** were first pretreated or not (control) for 15 min with 10 µM of LVV–hemorphin-7 and then stimulated 30 min with increasing doses of thrombin. Data are means ± SEM of three **(G** and **H)** or five to six independent experiments performed in triplicate. ****p < 0.0001, ***p < 0.001, **p < 0.01, and not statistically significant, *p > 0.05*.

Next, we examined the plausible effects of LVV–hemorphin-7 on AngII-mediated AT1R activation using dose–response as well as real-time kinetic BRET analysis. In dose–response experiments, cells were first pretreated for 15 min with different doses of LVV–hemorphin-7, and BRET signals were measured upon activation of AT1R with increasing doses of AngII. Interestingly, our results showed a significant left shift (more than one log, *p* < 0.001, n = 3) of the AngII curves for BRET between AT1R-Rluc and Venus-Gαq (**Figure 2A**) or yPET–β-arrestin 2 (**Figure 2B**) in cells pretreated with 10 or 100 µM of LVV–hemorphin-7 compared to control cells. In order to statistically consolidate our data, we replicated further our dose–response analysis in the absence (control) or presence of 10 µM of LVV–hemorphin-7. Indeed, our second set of data confirmed the nice left shift of the AngII dose curves for BRET between AT1R-Rluc and Venus-Gαq (*p* < 0.001 for the whole dose curve, n = 6) (**Figure 2C**) or yPET–β-arrestin 2 (*p* < 0.001 for the whole dose curve, n = 5) (**Figure 2D**) in cells pretreated with 10 µM of LVV–hemorphin-7 compared to control cells (**Table 1**). This demonstrated for the first time a positive effect of LVV–hemorphin-7 on AT1R activation transiently expressed in HEK293 cells. Based on these observations, a combination of 10 nM of AngII, leading to ∼25% of AT1R-mediated response, and 10 µM of LVV–hemorphin-7 was used in the different BRET kinetics and functional assays described below.

To further confirm the positive effect of LVV–hemorphin-7 on AT1R, we also performed dose–response experiments the other way around by preincubating or not cells for 15 min with increasing doses of LVV–hemorphin-7 followed by stimulation for 20 min with 10 nM of AngII. The data showed LVV–hemorphin-7 promoting a significant dose-dependent potentiation of AngII-mediated BRET increase between AT1R-Rluc and Venus-Gαq (**Figure 2E**) or yPET–β-arrestin 2 (**Figure 2F**) with EC_50_ in the micromolar range (**Table 1**).

As negative control to our data, we also tested the effect of LVV–hemorphin-7 on another unrelated GPCR, the thrombin receptor or protease-activated receptor 1 (PAR1). The data clearly showed no significant changes in the dose–response curves of thrombin-mediated BRET increase between PAR1-Rluc and either Venus-Gαq (**Figure 2G**) or yPET–β-arrestin 2 (**Figure 2H**).

### Real-Time BRET Kinetics of the Positive Effects of LVV–Hemorphin-7 on AT1R

**Figure 3:**
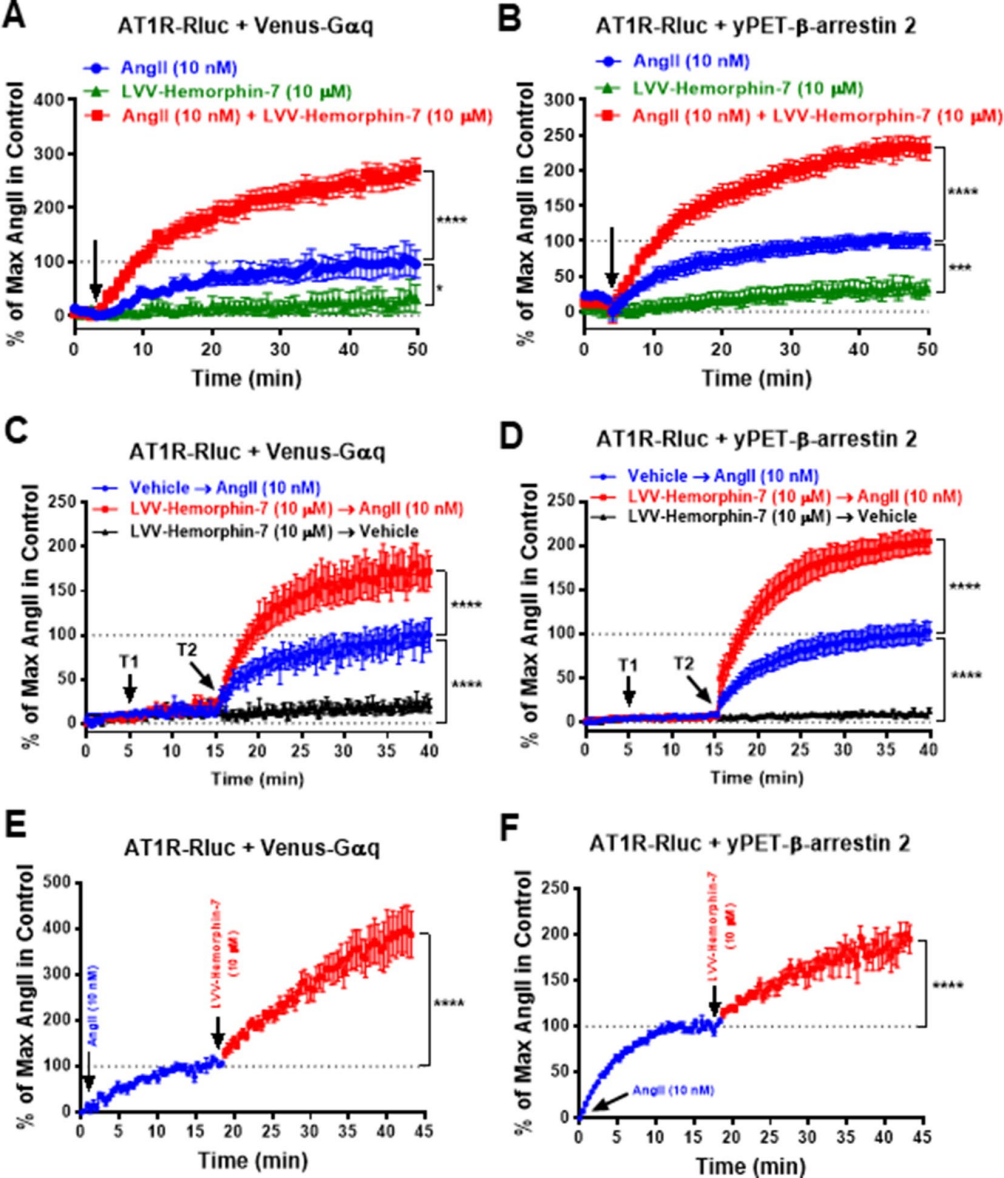
Positive effect of LVV–hemorphin-7 on AT1R activation revealed by real-time BRET kinetic analysis. HEK293 cells transiently coexpressing AT1R-Rluc and either Venus-Gαq **(A**, **C**, and **E)** or yPET–β-arrestin 2 **(B**, **D**, and **F)** were used for real-time BRET kinetic analysis as indicated in *Methods*. In **(A** and **B)**, BRET signals were measured before (baseline) and upon cell stimulation (arrow) with either 10 nM of AngII, 10 µM of LVV–hemorphin-7, or both during 50 min. In **(C** and **D)**, BRET signals were measured before any treatment (baseline) during 5 min and following treatment or not (vehicle) of cells with 10 µM of LVV–hemorphin-7 (T1) for 10 min and then additional treatment or not (vehicle) with 10 nM of AngII (T2) during 25 min. However, in **(E** and **F)**, cells were first stimulated with 10 nM of AngII, and BRET signals were measured for ∼20 min before an additional treatment with 10 µM of LVV–hemorphin-7 and further BRET measurements for 25 min. Data are means ± SEM of five to eight independent experiments performed in duplicate or triplicate. ****p < 0.0001, ***p < 0.001, *p < 0.05, and not statistically significant, *p > 0.05*.

To further characterize the positive action of LVV–hemorphin-7 on AT1R activation, real-time BRET kinetics were carried out in HEK293 cells transiently coexpressing AT1R-Rluc with either Venus-Gαq (**Figures 3A, C, E**) or yPET–β-arrestin 2 (**Figures 3B, D, F**) and treated with AngII and LVV–hemorphin-7 using different protocols and combinations. First, cells were straightway treated or not with either AngII (10 nM), LVV–hemorphin-7 (10 µM), or both, and BRET signals were measured in real time. The kinetics showed that the combination of AngII and LVV–hemorphin-7 strongly potentiated the BRET signals within AT1R-Rluc/Venus-Gαq (**Figure 3A**) as well as AT1R-Rluc/yPET–β-arrestin 2 (**Figure 3B**) pairs, compared to a single treatment of cells with AngII (**Table 2**). We also observed a partial agonistic effect of LVV–hemorphin-7 on BRET between AT1R-Rluc and yPET–β-arrestin 2 (**Figure 3B**) and to less extent between AT1R-Rluc and Venus-Gαq (**Figure 3A**).

**Table 2:** *E*_max_ values (%) of AngII in the different BRET assays.

Transfections	*E*_max_ (BRET dose–response)	*E*_max_ (BRET kinetics 1)	*E*_max_ (BRET kinetics 2)
AT1R-Rluc + Venus-Gαq	109 ± 2 (*p* > 0.05, n = 9)	263 ± 6 (*p* < 0.0001, n = 6)	171 ± 3 (*p* <0.0001, n = 8)
AT1R-Rluc + yPET–β-arrestin 2	104 ± 4 (*p* > 0.05, n = 8)	240 ± 5 (*p* < 0.0001, n = 5)	200 ± 0.2 (*p* < 0.0001, n = 5)

The statistical analysis is indicative of significance compared to the conditions with 10 nM AngII in the absence of LVV–hemorphin-7 (control) taken as 100%. Kinetics 1 and 2 refer to the data in **Figures 3A, B** and **Figures 3C, D**, respectively.

We also performed sequential real-time kinetics where BRET signals were measured before any cell treatment for 5 min, which positions the baseline level, followed by their treatment or not with 10 µM of LVV–hemorphin-7 (treatment 1 or T1) for 10 min, and finally by cell stimulation or not with 10 nM of AngII (treatment 2 or T2) for 25 min. The kinetics clearly showed a very weak (∼10%) BRET increase occurred in cells challenged with LVV–hemorphin-7 alone after 35 min of measurements. However, the AngII-induced BRET increase within AT1R-Rluc/Venus-Gαq (**Figure 3C**) and AT1R-Rluc/yPET–β-arrestin 2 (**Figure 3D**) pairs was strongly higher in cells previously treated with LVV–hemorphin-7 compared to those treated with vehicle (**Figures 3C, D**) (**Table 2**). Finally, BRET kinetics were also performed where cells were first stimulated with 10 nM of AngII for ∼15 to 20 min to promote BRET increase between AT1R-Rluc/Venus-Gαq (**Figure 3E**) and AT1R-Rluc/yPET–β-arrestin 2 (**Figure 3F**) pairs before adding 10 µM of LVV–hemorphin-7. The data showed a significant BRET potentiation beyond the AngII-induced level in both cases indicating the positive effect of LVV–hemorphin-7 can also be observed even after AngII binding to AT1R. Together, the kinetic observations are consistent with the dose–response experiments shown in **Figure 2** demonstrating the positive effect of LVV–hemorphin-7 on AT1R activation in HEK293 cells.

### LVV–Hemorphin-7 Effects on BRET Signals Involve the Activation of AT1R

**Figure 4:**
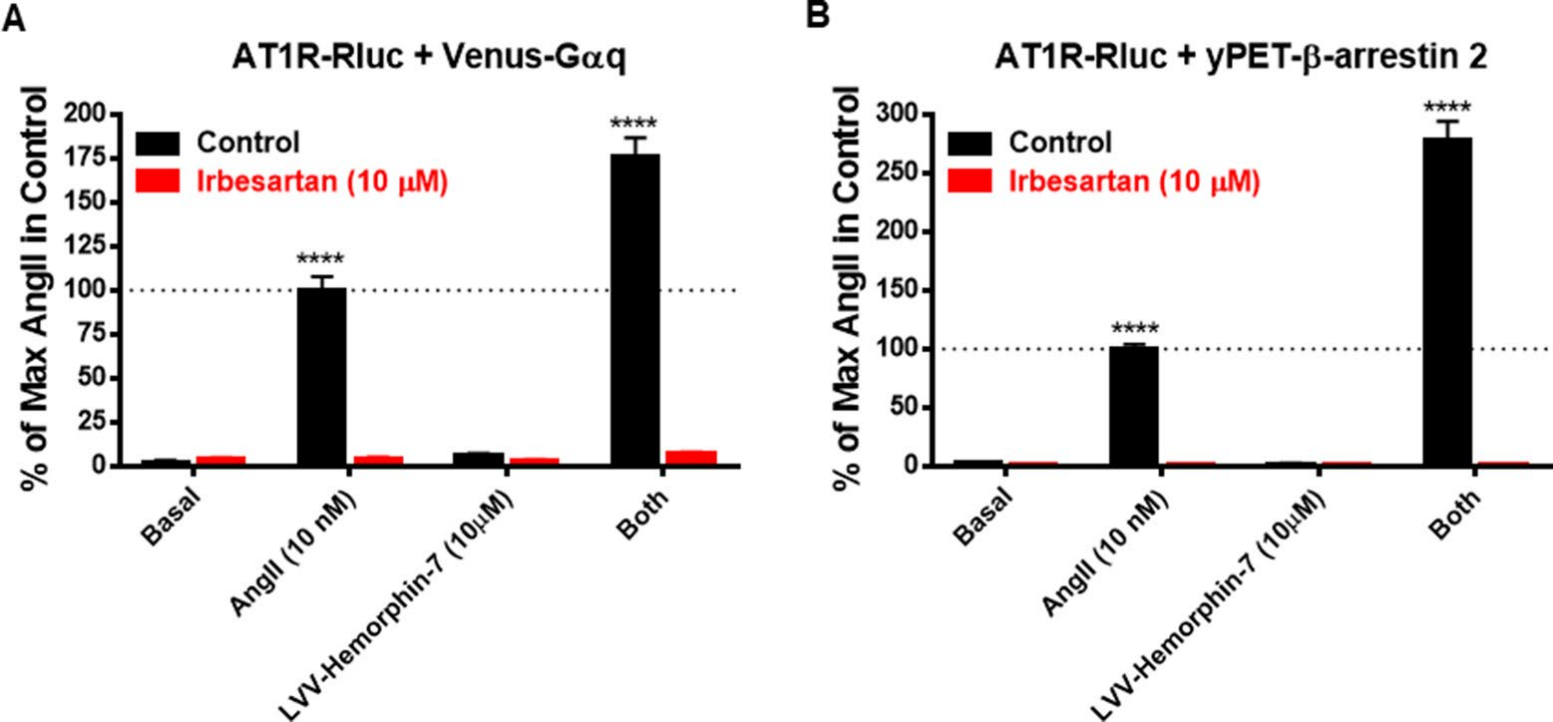
AT1R-selective antagonist blocked both AngII- and LVV–hemorphin-7–mediated BRET increase. HEK293 cells transiently coexpressing AT1R-Rluc and either Venus-Gαq **(A)** or yPET–β-arrestin 2 **(B)** were first pretreated or not (control using DMSO) with 10 µM of irbesartan for 15 min at 37ºC. Then, cells were stimulated or not (basal) with either 10 nM of AngII, 10 µM of LVV–hemorphin-7, or both, for 30 min at 37ºC before BRET measurements were performed as described in *Methods*. Data are means ± SEM of three independent experiments performed in duplicate. ****p < 0.0001, and not statistically significant, *p > 0.05*.

Next, we examined the effect of AT1R blockade on both AngII and LVV–hemorphin-7–mediated BRET increase. For this, we used irbesartan as a selective AT1R antagonist on HEK293 cells transiently coexpressing AT1R-Rluc with either Venus-Gαq (**Figure 4A**) or yPET–β-arrestin 2 (**Figure 4B**) treated or not with either AngII (10 nM), LVV–hemorphin-7 (10 µM), or both. Our data first confirmed the strong positive effects of LVV–hemorphin-7 on AngII-mediated BRET increase in both BRET configurations with AT1R-Rluc and either Venus-Gαq (**Figure 4A**) or yPET–β-arrestin 2 (**Figure 4B**). More importantly, we also observed a full inhibition of AngII-mediated BRET signals and their potentiation by LVV–hemorphin-7 upon treatment with 10 µM of irbesartan (**Figures 4A, B**). These data clearly demonstrate that the positive action of LVV–hemorphin-7 involves the active conformation of AT1R.

### The Positive Effects of LVV–Hemorphin-7 on AT1R-Mediated Downstream Signaling Pathways

**Figure 5:**
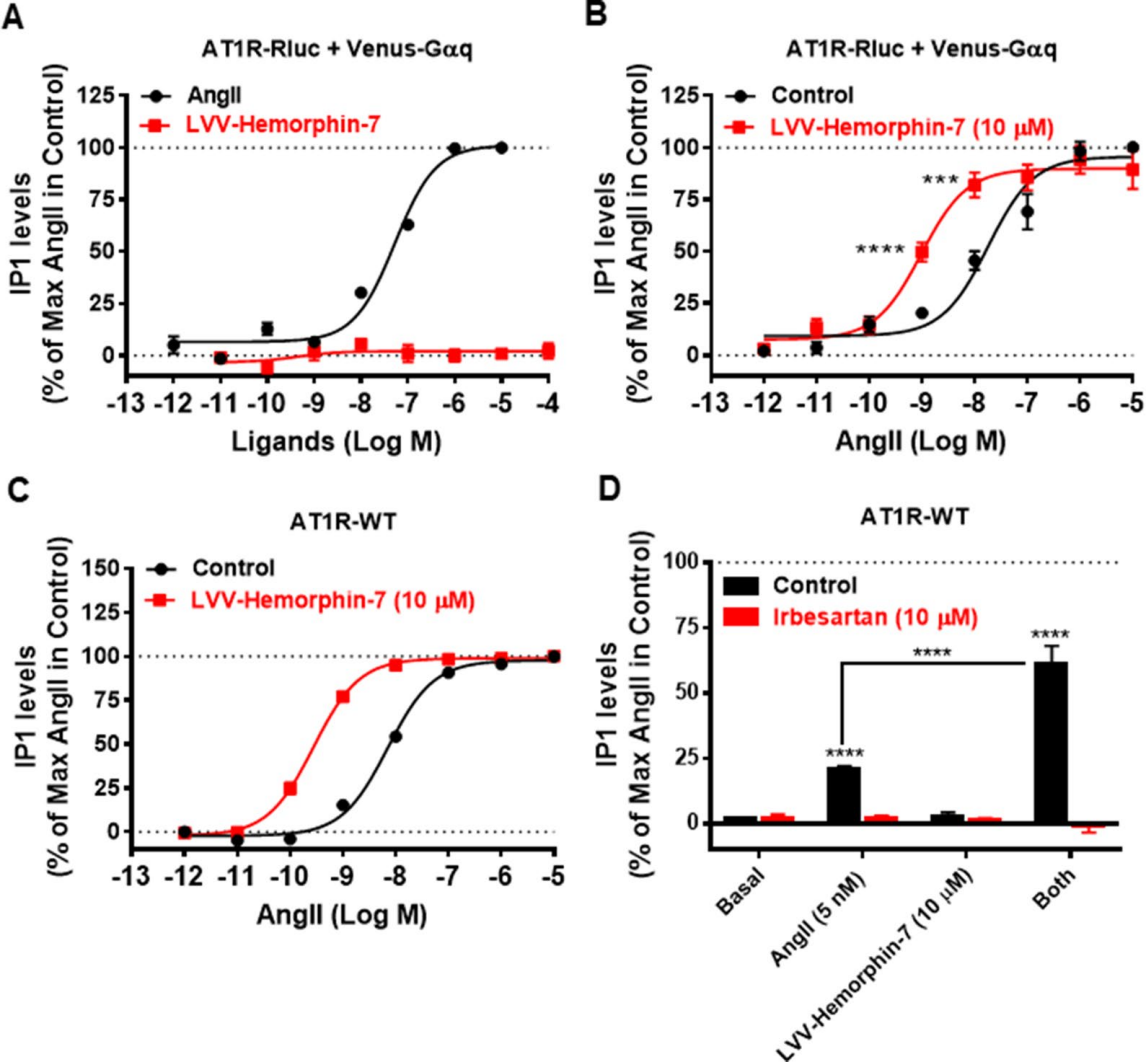
Positive effect of LVV–hemorphin-7 on AT1R-mediated IP1 production. HEK293 cells transiently coexpressing AT1R-Rluc with Venus-Gαq **(A** and **B)** or AT1R-WT **(C** and **D)** were used for IP1 assays. For the dose–response experiments, cells were stimulated or not with the increasing doses of either AngII or LVV–hemorphin-7 **(A)** or first pretreated or not (control) with 10 µM of LVV–hemorphin-7 for 15 min and then stimulated 30 min with increasing doses of AngII **(B** and **C)** before IP1 measurements as described in *Methods*. In **(D)**, cells expressing AT1R-WT were first pretreated or not (control using DMSO) with 10 µM of irbesartan for 15 min at 37ºC. Then, cells were stimulated or not (basal) with either 5 nM of AngII, 10 µM of LVV–hemorphin-7, or both, for 30 min at 37ºC before IP1 measurements were performed as described in *Methods*. IP1 data are means ± SEM of three to seven independent experiments performed in duplicate or triplicate. ****p < 0.0001, ***p < 0.001, and not statistically significant, *p > 0.05*.

To correlate our BRET data with the downstream signaling pathways of AT1R, we examined the effects of LVV–hemorphin-7 on AT1R-mediated IP1 production as a readout for Gαq/phospholipase C activation and ERK1/2 phosphorylation. For this, we first used HEK293 cells transiently coexpressing AT1R-Rluc and Venus-Gαq in order to use similar experimental conditions as for BRET assays. First, we tested the putative agonistic effect of LVV–hemorphin-7 on AT1R-mediated IP1 production in cells stimulated with increasing doses of either AngII as control or LVV–hemorphin-7. As shown in **Figure 5A**, while AngII promoted IP1 production in a dose-dependent manner and with the expected potency (**Table 1**), LVV–hemorphin-7 had no significant effect even at 100 µM. Next, we examined the positive effect of LVV–hemorphin-7 on AngII-mediated IP1 response by stimulating cells with the increasing doses of AngII in the absence or presence of pretreatment of cells with 10 µM of LVV–hemorphin-7 for 15 min. As shown in **Figure 5B**, like in BRET assays, LVV–hemorphin-7 promoted a significant left shift of the AngII dose-dependent curve with a stronger (∼18.6-fold increase in AngII EC_50_) effect compared to BRET data (**Table 1**).

To rule out any artifact in our results due to the utilization of AT1R-Rluc coexpressed with Venus-Gαq similarly to BRET assays, we also performed IP1 experiments in HEK293 cells transiently expressing the untagged AT1R (AT1R-WT). As shown in **Figure 5C**, AngII nicely induced IP1 production in a dose–response manner, and cotreatment of cells with 10 µM of LVV–hemorphin-7 significantly left shifted the AngII dose-dependent curve with ∼25-fold increase in AngII EC_50 _(**Table 1**). This further supports the positive effect of LVV–hemorphin-7 on AT1R-mediated IP1 response.

Furthermore, we examined the effect of AT1R blockade using its selective antagonist, irbesartan, similarly to BRET assays. For this, we used AT1R-WT expressing HEK293 cells pretreated or not with10 µM of irbesartan followed by treatment or not with 10 µM of LVV–hemorphin-7 combined or not with a nonsaturating dose of AngII (5 nM). Notice that 5 nM of AngII in IP1 on AT1R-WT corresponds to 10 nM used in BRET assays shown in **Figure 4**, and this was determined based on the dose curve obtained on AT1R-WT shown in **Figure 5C**. As shown in **Figure 5D**, both AngII-mediated IP1 production and its potentiation by LVV–hemorphin-7 were completely abolished by irbesartan treatment. These data are consistent with the BRET data shown in **Figure 4** and further demonstrate the engagement of the active conformation of AT1R is such pharmacological effects of LVV–hemorphin-7 on AngII-mediated IP1 response.

**Figure 6:**
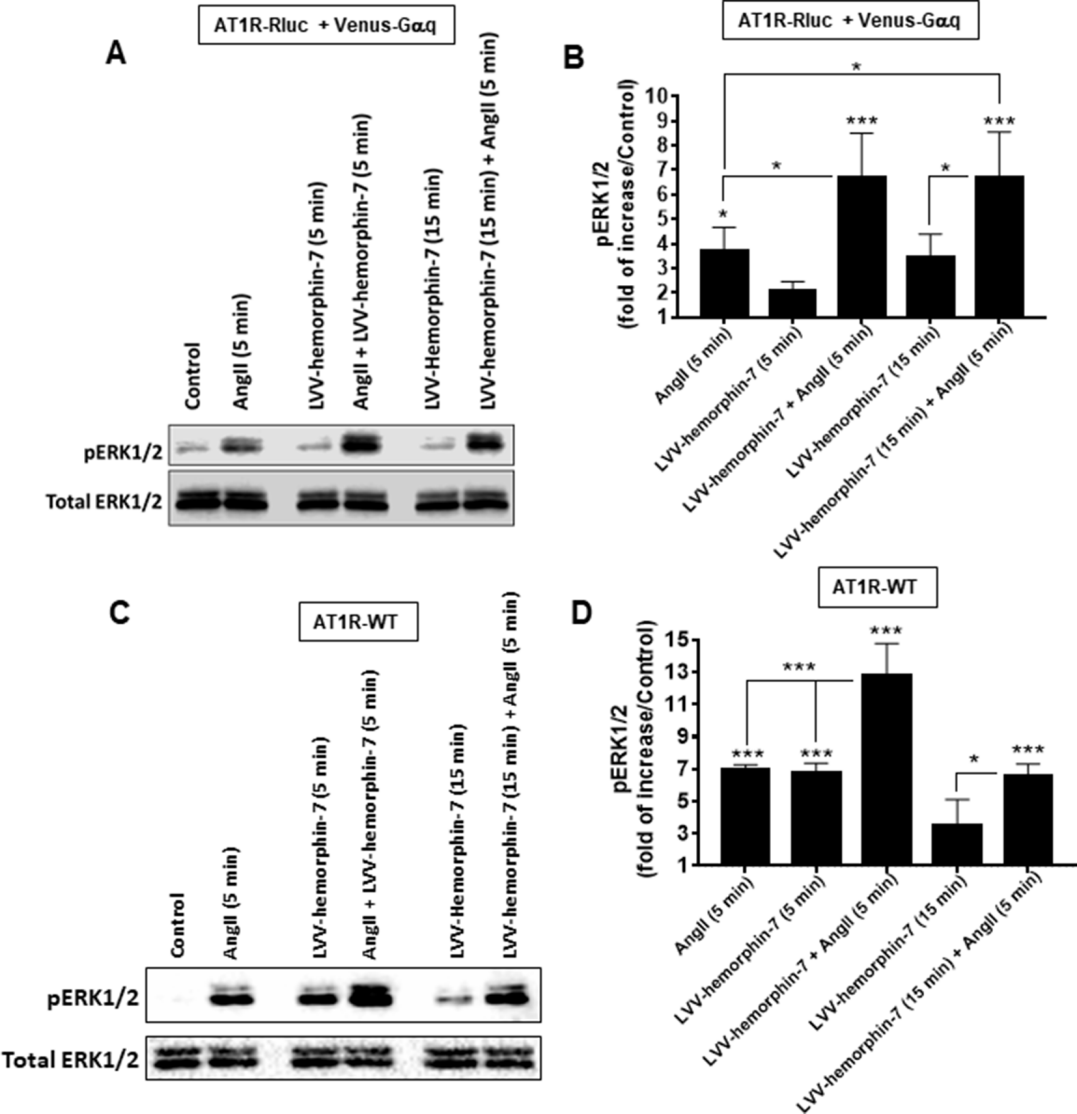
Positive effect of LVV–hemorphin-7 on AT1R-mediated ERK1/2 phosphorylation. HEK293 cells transiently coexpressing AT1R-Rluc with Venus-Gαq **(A** and **B)** or AT1R-WT **(C** and **D)** were used for ERK1/2 experiments. For this, cells were stimulated 5 or 15 min with 10 nM of AngII, 10 µM of LVV–hemorphin-7, or both, as indicated, and SDS-PAGE followed by Western blot for the phosphorylated as well as total ERK1/2 was carried out and described in *Methods*. The representative experiment for each transfection is shown in **(A** and **C)**. **(B** and **D)** represent the quantification of Western blot data for phospho-ERK1/2 changes using densitometry analysis, and the bars are means ± SEM of four independent experiments. The stars represent the statistical significance relative to the control condition (untreated cells), as well as the significance between the different treatments as indicated. ***p < 0.001, *p < 0.05, and not statistically significant, *p > 0.05*.

Finally, we examined AT1R-mediated ERK1/2 phosphorylation in HEK293 cells expressing either AT1R-Rluc with Venus-Gαq to use similar conditions as for BRET assays (**Figures 6A, B**) or AT1R-WT to examine the effects on the unmodified receptor (**Figures 6C, D**). For this, SDS-PAGE followed by Western blot was carried out on lysates from cells pretreated for 15 min or not with 10 µM of LVV–hemorphin-7 and then stimulated or not for 5 min with either 10 nM of AngII, 10 µM of LVV–hemorphin-7, or both. In cells coexpressing AT1R-Rluc with Venus-Gαq, the results showed a significant ERK1/2 phosphorylation promoted by AngII (3.70 ± 0.95 folds, n = 4) and by LVV–hemorphin-7 (2.05 ± 0.38 folds, n = 4) at both 5 and 15 min (3.42 ± 0.97 folds, n = 4) compared to untreated cells (control) (**Figures 6A, B**). More interestingly, the cotreatment of cells with AngII and LVV–hemorphin-7 for 5 min strongly potentiated ERK1/2 phosphorylation (6.71 ± 1.78 folds, n = 4) compared to the individual treatment with AngII or LVV–hemorphin-7 (**Figures 6A, B**). Such a potentiation was also observed upon pretreatment with LVV–hemorphin-7 for 15 min followed by AngII stimulation for 5 min (6.70 ± 1.84 folds, n = 4).

A similar pattern of ERK1/2 phosphorylation was observed in HEK293 cells expressing AT1R-WT with both AngII and LVV–hemorphin-7 significantly increasing ERK1/2 phosphorylation (6.94 ± 0.31 folds, n = 4) (**Figures 6C, D**). In addition, the cotreatment of cells with AngII and LVV–hemorphin-7 strongly potentiated the response (12.75 ± 2.01 folds, n = 4) compared to the individual treatments with either AngII or LVV–hemorphin-7 (**Figures 6C, D**). Interestingly, in AT1R-WT expressing cells LVV–hemorphin-7 at 5 min induced a strong agonistic effect similarly to AngII (6.74 ± 0.62 folds, n = 4) (**Figures 6C, D**).

Together, IP1 and ERK1/2 data using both AT1R-Rluc and AT1R-WT further demonstrate the positive effect of LVV–hemorphin-7 on AT1R activation and its downstream signaling. Moreover, the ERK1/2 data clearly suggest an agonistic action of LVV–hemorphin-7 on AT1R in addition to its potentiating effect on AngII-mediated ERK1/2 response.

## Discussion

In this study, we report for the first time the pharmacological action of LVV–hemorphin-7 on AT1R transiently expressed in HEK293 cells with an interesting positive action on AT1R activation and signaling. This was demonstrated using *in vitro* assays including real-time BRET for AngII-mediated AT1R-Gαq coupling as well as β-arrestin 2 recruitment as well as their related downstream signaling pathways through the measurements of IP1 production and ERK1/2 phosphorylation. Indeed, our BRET data clearly demonstrate the positive effects of LVV–hemorphin-7 on AT1R activation and its functional coupling with Gαq and its interaction with β-arrestin 2. Both dose–response and real-time kinetics revealed a strong potentiation of AngII-induced BRET signals in the presence of LVV–hemorphin-7. Such a positive action was also observed on AngII-mediated Gαq/IP1 production and ERK1/2 phosphorylation with an interesting agonistic effect of LVV–hemorphin-7 on ERK1/2 response revealed on both AT1R-Rluc and AT1R-WT expressing cells. Thus, our data suggest a differential action of LVV–hemorphin-7 on AT1R-mediated signaling with a positive allosteric modulation (PAM)-like effect on Gαq activation and β-arrestin 2 recruitment (assessed by BRET) and an agonistic action on ERK1/2 phosphorylation. To find whether this is indicative of biased effects of LVV–hemorphin-7 on AT1R or not, further investigation is required.

Our seminal finding is of significant importance to hemorphin and GPCR pharmacology and their implication in human physiology. Indeed, in addition to hemorphin’s effect on opioid receptors (Brantl et al., 1986; Davis et al., 1989; Liebmann et al., 1989; Glämsta et al., 1992; Yukhananov et al., 1994; Nyberg et al., 1997; Szikra et al., 2001; Cheng et al., 2012), we report yet another GPCR member (AT1R) that is a target of hemorphins with the plausible implication in the physiology and pathophysiology of vascular and renal systems. The action of LVV–hemorphin-7 on AT1R is consistent with the previous observations demonstrating LVV–hemorphin-7 acting on opioid receptors extending the spectra of action of hemorphins on GPCRs. For the opioid receptors, a partial to full radioligand binding was reported with a competition with some endogenous enkephalin- and dynorphin-related opioid peptides but not nonpeptide ligands (Garreau et al., 1995; Zhao and Piot, 1997; Szikra et al., 2001). Moreover, LVV–hemorphin-6 (Yukhananov et al., 1994) and -7 (Szikra et al., 2001) showed agonist-like effect as demonstrated in guinea-pig ileum and GTPγS binding assays, respectively. This clearly demonstrated the binding of these hemorphins on the opioid receptors that explain the analgesic effects of hemorphin observed *in vivo* (Hughes et al., 1975; Moisan et al., 1998; Sanderson et al., 1998; Albiston et al., 2004; Cejka et al., 2004; Lee et al., 2004; Cheng et al., 2012). Of course, our data do not prove a direct binding of LVV–hemorphin-7 on AT1R, but the strong positive effects observed in IP1 and BRET assays, as well as the agonistic action of LVV–hemorphin-7, observed in ERK1/2 assay and to very less extent BRET (100 µM), suggest such an interaction between LVV–hemorphin-7 and AT1R. Therefore, our speculation on the pharmacological effects of LVV–hemorphin-7 on AT1R emphasizes various possibilities and scenarios. First, our IP1 and BRET dose–response curves are typical of PAM of LVV–hemorphin-7 on AT1R with no significant effects of LVV–hemorphin-7 when applied alone and a nice left shift of the AngII dose curves in the presence of increasing doses of LVV–hemorphin-7. Of course, this implies that AT1R can be subjected to allosteric modulation and may present an allosteric binding site as suggested in many previous studies. For instance, an old study by Boulay et al. (1992) reported a negative allosteric modulation of AT1R by polyvinyl sulfate characterized by an inhibition of AngII affinity. Recently, PAM of AT1R by mechanical stretch of the cells has been reported (Tang et al., 2014; Wang et al., 2018). Moreover, another recent study reported a PAM of AT1R by homocysteine inducing an aggravation of the vascular injury (Li et al., 2018). Interestingly, the binding experiments showed in this study indicated the binding of homocysteine to the orthosteric binding site of AT1R along with AngII with a putative allosteric interaction between both within the binding pocket (Li et al., 2018). More recently, an allosteric AT1R-selective nanobody has been developed and used to stabilize the active conformation of AT1R and to crystalize it (Wingler et al., 2019). All these studies indicate the susceptibility of AT1R to be allosterically modulated by different kind of molecules (amino acids, nonpeptides, mechanical), and our study extends this feature to peptides (hemorphins) as potential allosteric modulators of AT1R. The other possibility to explain our data is the targeting of AT1R dimers/oligomers. In such a scenario, it is possible that no allosteric binding site exists, and only binding to the orthosteric site in AT1R occurred. This implies that both LVV–hemorphin-7 and AngII might bind in the orthosteric binding sites on two AT1R protomers, resulting in allosteric communication and effects within the AT1R dimers that lead to an increase in AngII binding and/or potency and efficacy. The putative binding of LVV–hemorphin-7 in the orthosteric binding site of AT1R would be consistent with the recent study on the allosteric modulation of AT1R by homocysteine showing binding of homocysteine to the orthosteric binding site of AT1R along with AngII with a putative allosteric interaction between both within the binding pocket (Li et al., 2018). In our study, ERK1/2 experiments clearly showed an agonistic action of LVV–hemorphin-7 on AT1R similarly to AngII, suggesting the possible implication of AT1R orthosteric binding site. Thus, we cannot exclude that ERK1/2 data reflect LVV–hemorphin-7 targeting AT1R dimers. Furthermore, previous studies showed binding of LVV–hemorphin-7 on opioid receptors partially to fully compete with the binding of some endogenous enkephalin- and dynorphin-related opioid peptides but not nonpeptide ligands (Garreau et al., 1995; Zhao and Piot, 1997; Szikra et al., 2001). Moreover, LVV–hemorphin-6 and -7 showed agonist-like effect and GTPγS binding assays (Yukhananov et al., 1994; Szikra et al., 2001). This suggests the binding of hemorphins in the orthosteric binding site of the opioid receptors without excluding the existence of another nonopioid binding site. Moreover, such a PAM of GPCRs involving effects on homodimers/heterodimers is a very important and exciting topic, and it has been reported in many occasions (Pin et al., 2005; Christopoulos, 2014; Liu et al., 2017). Finally, we cannot exclude an indirect effect of LVV–hemorphin-7 on AT1R through binding to another membrane AT1R-interacting protein expressed in HEK293 cells, resulting in transactivation of AT1R upon AngII binding. Further structural and functional studies are being considered to fully understand the exact mechanism of LVV–hemorphin-7 action on AT1R and its modulation. The profiling of other forms of hemorphin peptides should highlight further structure–function relationship data.

**Figure 7:**
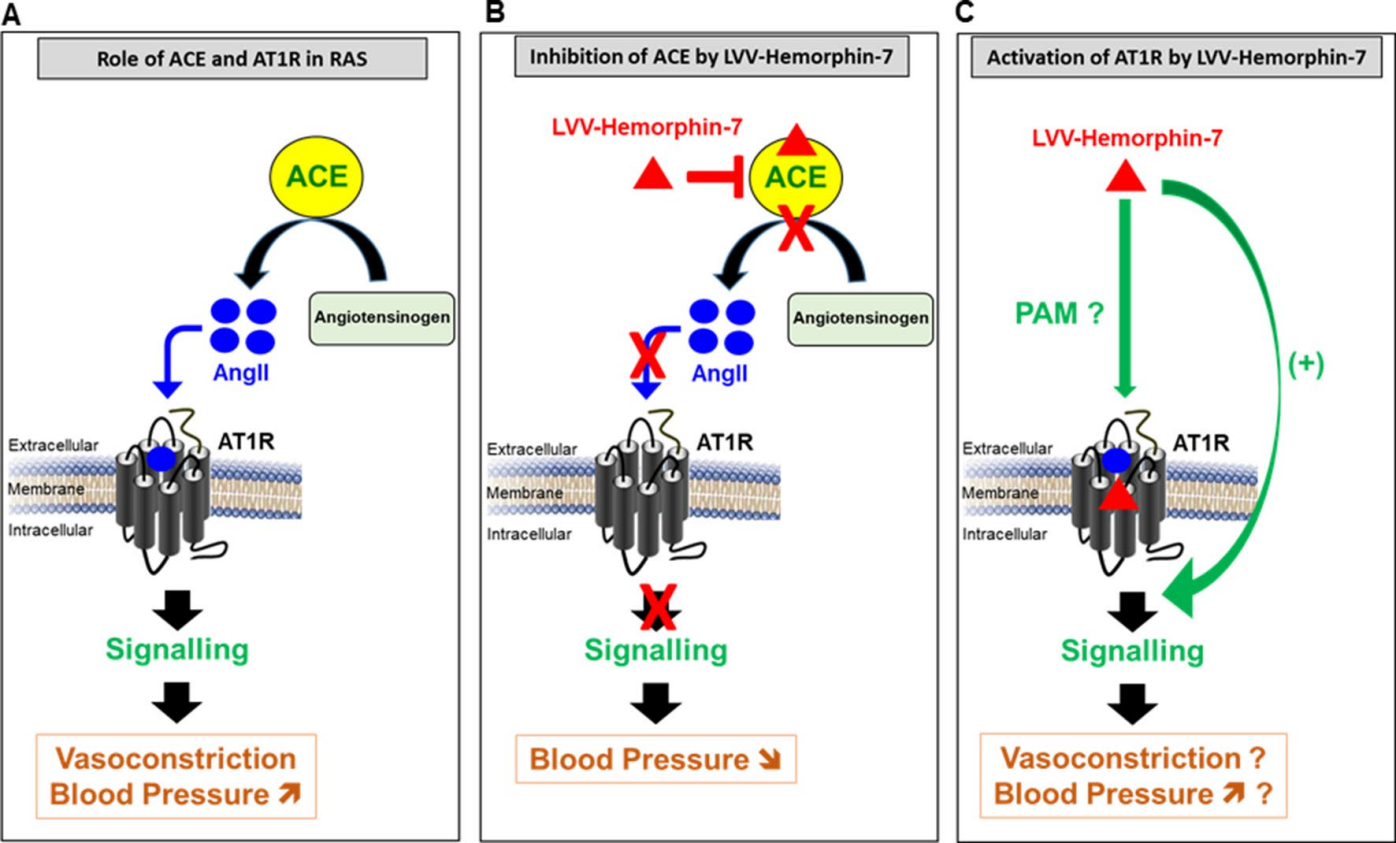
Model describing the pivotal role of AT1R and ACE in RAS **(A)** and the dual pharmacological effect of LVV–hemorphin-7 on these two major RAS components, inhibition of ACE **(B)** and activation of AT1R-mediated signaling **(C)** with a putative implication in the regulation of blood pressure.

From the physiological point of view, the positive effects of LVV–hemorphin-7 on AT1R interestingly suggest a hypertensive action. This is somehow inconsistent with the antihypertensive effects of hemorphins reported in many *in vitro* and *in vivo* studies (**Figure 7**) (Lantz et al., 1991; Zhao and Piot, 1997; Fruitier-Arnaudin et al., 2002; Cejka et al., 2004; Ianzer et al., 2006; Dejouvencel et al., 2010). For instance, hemorphins have been shown to produce antihypertensive effects *via* the inhibition of ACE (Zhao and Piot, 1997; Fruitier-Arnaudin et al., 2002). Moreover, hemorphins have been shown to increase the hypotensive effect of bradykinin (Ianzer et al., 2006). Finally, serum hemorphin-7 levels were found to be very high after long-distance running (Glämsta et al., 1993), whereas they were very low in obese and diabetic patients, which are linked to hypertension and cardiovascular risks (Maraninchi et al., 2013). The contrasting effects of hemorphins on AT1R (activation) and ACE (inhibition) and their physiological consequences, hypertensive and hypotensive, respectively, illustrate the complexity of hemorphin’s action on RAS. One way to reconcile these contrasting effects is by speculating that the inhibition of ACE that leads to a reduction of AngII levels may be counterbalanced by the positive action of hemorphins on AT1R. This would imply that, in this condition, low doses of AngII might be sufficient to activate RAS. Furthermore, one could argue that hemorphins control blood pressure and water reabsorption under various circumstances by biasing RAS toward activation through their positive effects on AT1R or inhibition of ACE activity and AngII levels. From our point of view, our data can be interpreted within both physiological and pathophysiological contexts. Indeed, it is not really clear whether the release of hemorphins from the hemoglobin constitutes a physiological or a pathogenic process with an impact on blood pressure and water balance. Moreover, it is not clear which component, hemorphins, AT1R, or ACE, is really the determinant one in the system and how this is involved in both physiological and pathophysiological situations. In the physiological situation, the dual effect of hemorphins may be part of the fine regulation of the blood pressure (and maybe water balance as well) through the positive and the negative actions on AT1R and ACE, respectively, in order to keep the blood pressure within the normal ranges. For instance, one would argue that if high blood pressure occurs for any reason and this might be associated with high expression and/or activity of ACE, hemorphins might be then released to counterbalance this situation in order to avoid any risk of sustained or chronic hypertension. In the same line, if a hypotensive/vasodilatation situation comes to occur because of low expression/activity of AT1R, hemorphins may be then released to increase blood pressure by promoting AT1R-dependent vasoconstriction. In the case of a pathological situation, the high expression/activity of AT1R associated with a plausible decrease in ACE expression/activity and the high release of hemorphins may be symptomatic of the pathophysiology of RAS and hypertension. In that case, hypertension might be either the reason or the consequence of hemorphin’s action. Thus, we believe that the system involving all the three components (hemorphins, AT1R, or ACE) may depend on the circulating concentrations of hemorphins along with the relative expression levels of the two targets, ACE and AT1R, in the different physiological and pathophysiological circumstances. So far, there are no reliable data on the plausible relationship between the concentrations of hemorphins released in blood and the expression profiles of AT1R and ACE and its impact on the control of blood pressure and hypertension. This implies that the stoichiometry hemorphins/AT1R versus hemorphins/ACE is not completely clear under hypotensive and hypertensive situations. In one old study, it has been reported that long-distance running increased the blood content in hemorphins (Glämsta et al., 1993). This may be consistent with either a positive action of hemorphins (hypertensive) through targeting AT1R because of the exercise or their negative regulatory effect (hypotensive) through the inhibition of ACE to avoid any excessive hypertension. In addition, very low hemorphin levels were recently reported in obese and diabetic patients that are linked to hypertension and cardiovascular risks suggesting beneficial effects of hemorphins on the inhibition of ACE (Maraninchi et al., 2013). Finally, it would be interesting to investigate the plausible effects of hemorphins on other key actors of RAS and kidney such as adrenergic, bradykinin, and vasopressin receptors, known to have functional interactions with AT1R (Tóth et al., 2018).

## Data Availability Statement

All datasets generated for this study are included in the manuscript.

## Author Contributions

AAl, AP, AAs, IA, and MA performed the research and analyzed the data; BB analyzed the data; MA and RV conceived the project, wrote the manuscript, and managed the project and its funding.

## Funding

This work was supported by the United Arab Emirates University startup grant (31S305) to MA and a UPAR grant (31S243) to RV.

## Conflict of Interest

The authors declare that the research was conducted in the absence of any commercial or financial relationships that could be construed as a potential conflict of interest.

## References

[B1] Albiston A. L. Pederson E. S. Burns P. Purcell B. Wright J. W. Harding J. W. (2004). Attenuation of scopolamine-induced learning deficits by LVV-hemorphin-7 in rats in the passive avoidance and water maze paradigms. Behav. Brain Res. 154 (1), 239–243. 10.1016/j.bbr.2004.02.012 15302130

[B2] Ayoub M. A. (2016). Resonance Energy Transfer-Based Approaches to Study GPCRs. Methods Cell Biol 132, 255–292. 10.1016/bs.mcb.2015.10.008 26928548

[B3] Ayoub M. A. Landomiel F. Gallay N. Jégot G. Poupon A. Crépieux P. (2015). Assessing gonadotropin receptor function by resonance energy transfer-based assays. Front. Endocrinol. 6, 1–14. 10.3389/fendo.2015.00130 PMC4550792 26379624

[B4] Ayoub M. A. Yvinec R. Jégot G. Dias J. A. Poli S.-M. Poupon A. (2016). Profiling of FSHR negative allosteric modulators on LH/CGR reveals biased antagonism with implications in steroidogenesis. Mol. Cell. Endocrinol. 436, 10–22. 10.1016/j.mce.2016.07.013 27424143

[B5] Bonde M. M. Hansen J. T. Sanni S. J. Haunsø S. Gammeltoft S. Lyngsø C. (2010). Biased signaling of the angiotensin II type 1 receptor can be mediated through distinct mechanisms. PLoS One 5 (11), e14135. 10.1371/journal.pone.0014135 PMC2994726 21152433

[B6] Boulay G. Servant G. Luong T. T. Escher E. Guillemette G. (1992). Modulation of angiotensin II binding affinity by allosteric interaction of polyvinyl sulfate with an intracellular domain of the DuP-753-sensitive angiotensin II receptor of bovine adrenal glomerulosa. Mol. Pharmacol. 41 (4), 809–815. 1569928

[B7] Brantl V. Gramsch C. Lottspeich F. Mertz R. Jaeger K. H. Herz A. (1986). Novel opioid peptides derived from hemoglobin: hemorphins. Eur. J. Pharmacol. 125 (2), 309–310. 10.1016/0014-2999(86)90044-0 3743640

[B8] Cejka J. Zelezná B. Velek J. Zicha J. Kunes J. (2004). LVV-hemorphin-7 lowers blood pressure in spontaneously hypertensive rats: radiotelemetry study. Physiol. Res. 53 (6), 603–607. 15588127

[B9] Cheng B.-C. Tao P.-L. Cheng Y.-Y. Huang E. Y.-K. (2012). LVV-hemorphin 7 and angiotensin IV in correlation with antinociception and anti-thermal hyperalgesia in rats. Peptides 36 (1), 9–16. 10.1016/j.peptides.2012.03.019 22484286

[B10] Chow L.-H. Chen Y.-H. Lai C.-F. Lin T.-Y. Chen Y.-J. Kao J.-H. (2018). Sex difference of angiotensin IV-, LVV-hemorphin 7-, and oxytocin-induced antiallodynia at the spinal level in mice with neuropathic pain. Anesth. Analg. 126 (6), 2093–2101. 10.1213/ANE.0000000000002795 29381512

[B11] Christopoulos A. (2014). Advances in G protein-coupled receptor allostery: from function to structure. Mol. Pharmacol. 86 (5), 463–478. 10.1124/mol.114.094342 25061106

[B12] Dagouassat N. Garreau I. Zhao Q. Sannier F. Piot J. M. (1996). Kinetic of *in vitro* generation of some hemorphins: early release of LVV-hemorphin-7, precursor of VV-hemorphin-7. Neuropeptides 30 (1), 1–5. 10.1016/S0143-4179(96)90047-5 8868292

[B13] Davis T. P. Gillespie T. J. Porreca F. (1989). Peptide fragments derived from the beta-chain of hemoglobin (hemorphins) are centrally active *in vivo* . Peptides 10 (4), 747–751. 10.1016/0196-9781(89)90107-1 2587417

[B14] Dejouvencel T. Féron D. Rossignol P. Sapoval M. Kauffmann C. Piot J.-M. (2010). Hemorphin 7 reflects hemoglobin proteolysis in abdominal aortic aneurysm. Arterioscler. Thromb. Vasc. Biol. 30 (2), 269–275. 10.1161/ATVBAHA.109.198309 19910633

[B15] Domazet I. Holleran B. J. Richard A. Vandenberghe C. Lavigne P. Escher E. (2015). Characterization of angiotensin II molecular determinants involved in AT1 receptor functional selectivity. Mol. Pharmacol. 87 (6), 982–995. 10.1124/mol.114.097337 25808928

[B16] Erchegyi J. Kastin A. J. Zadina J. E. Qiu X. D. (1992). Isolation of a heptapeptide Val-Val-Tyr-Pro-Trp-Thr-Gln (valorphin) with some opiate activity. Int. J. Pept. Protein Res. 39 (6), 477–484. 10.1111/j.1399-3011.1992.tb00277.x 1356941

[B17] Fruitier-Arnaudin I. Cohen M. Bordenave S. Sannier F. Piot J.-M. (2002). Comparative effects of angiotensin IV and two hemorphins on angiotensin-converting enzyme activity. Peptides 23 (8), 1465–1470. 10.1016/S0196-9781(02)00083-9 12182948

[B18] Garreau I. Zhao Q. Pejoan C. Cupo A. Piot J. M. (1995). VV-hemorphin-7 and LVV-hemorphin-7 released during *in vitro* peptic hemoglobin hydrolysis are morphinomimetic peptides. Neuropeptides 28 (4), 243–250. 10.1016/0143-4179(95)90028-4 7596489

[B19] Glämsta E. L. Meyerson B. Silberring J. Terenius L. Nyberg F. (1992). Isolation of a hemoglobin-derived opioid peptide from cerebrospinal fluid of patients with cerebrovascular bleedings. Biochem. Biophys. Res. Commun. 184 (2), 1060–1066. 10.1016/0006-291X(92)90699-L 1575724

[B20] Glämsta E. L. Mørkrid L. Lantz I. Nyberg F. (1993). Concomitant increase in blood plasma levels of immunoreactive hemorphin-7 and beta-endorphin following long distance running. Regul Pept. 49 (1), 9–18. 10.1016/0167-0115(93)90378-L 7904083

[B21] Hughes J. Smith T. W. Kosterlitz H. W. Fothergill L. A. Morgan B. A. Morris H. R. (1975). Identification of two related pentapeptides from the brain with potent opiate agonist activity. Nature 258 (5536), 577–580. 10.1038/258577a0 1207728

[B22] Hunyady L. Catt K. J. (2006). Pleiotropic AT1 receptor signaling pathways mediating physiological and pathogenic actions of angiotensin II. Mol. Endocrinol. 20, 953–970. 10.1210/me.2004-0536 16141358

[B23] Ianzer D. Konno K. Xavier C. H. Stöcklin R. Santos R. A. S. de Camargo A. C. M. (2006). Hemorphin and hemorphin-like peptides isolated from dog pancreas and sheep brain are able to potentiate bradykinin activity *in vivo.* Peptides 27 (11), 2957–2966. 10.1016/j.peptides.2006.06.009 16904236

[B24] Inagami T. (1999). Molecular biology and signaling of angiotensin receptors: an overview. J. Am. Soc. Nephrol. 10 Suppl 11, S2–S7. 9892133

[B25] Ivanov V. T. Karelin A. A. Philippova M. M. Nazimov I. V. Pletnev V. Z. (1997). Hemoglobin as a Source of Endogenous Bioactive Peptides: the Concept of Tissue-Specific Peptide Pool. Biopolymers 43 (2), 171–188. 10.1002/(SICI)1097-0282(1997)43:2<171::AID-BIP10>3.0.CO;2-O 9216253

[B26] Johnston C. I. (1992). Franz Volhard Lecture. Renin-angiotensin system: a dual tissue and hormonal system for cardiovascular control. J. Hypertens. Suppl. 10, S13–S26. 10.1097/00004872-199212000-00002 1337911

[B27] Karelin A. A. Philippova M. M. Karelina E. V. Ivanov V. T. (1994). Isolation of endogenous hemorphin-related hemoglobin fragments from bovine brain. Biochem. Biophys. Res. Commun. 202 (1), 410–415. 10.1006/bbrc.1994.1943 8037741

[B28] Lantz I. Glämsta E. L. Talbäck L. Nyberg F. (1991). Hemorphins derived from hemoglobin have an inhibitory action on angiotensin converting enzyme activity. FEBS Lett. 287 (1–2), 39–41. 10.1016/0014-5793(91)80011-Q 1652464

[B29] Lee J. Albiston A. L. Allen A. M. Mendelsohn F. A. O. Ping S. E. Barrett G. L. . (2004). Effect of I.C.V. injection of AT4 receptor ligands, NLE1-angiotensin IV and LVV-hemorphin 7, on spatial learning in rats. Neuroscience 124 (2), 341–349. 10.1016/j.neuroscience.2003.12.006 14980384

[B30] Li T. Yu B. Liu Z. Li J. Ma M. Wang Y. (2018). Homocysteine directly interacts and activates the angiotensin II type I receptor to aggravate vascular injury. Nat. Commun. 9 (1), 11. 10.1038/s41467-017-02401-7 29296021 PMC5750214

[B31] Liebmann C. Schrader U. Brantl V. (1989). Opioid receptor affinities of the blood-derived tetrapeptides hemorphin and cytochrophin. Eur. J. Pharmacol. 166 (3), 523–526. 10.1016/0014-2999(89)90368-3 2553436

[B32] Liu J. Zhang Z. Moreno-Delgado D. Dalton J. A. Rovira X. Trapero A. (2017). Allosteric control of an asymmetric transduction in a G protein-coupled receptor heterodimer. Elife 10, 6. 10.7554/eLife.26985 PMC5582870 28829739

[B33] Maraninchi M. Feron D. Fruitier-Arnaudin I. Bégu-Le Corroller A. Nogueira J. P. Mancini J. (2013). Serum hemorphin-7 levels are decreased in obesity. Obesity (Silver Spring). 21 (2), 378–381. 10.1002/oby.20280 23532992

[B34] Moeller I. Lew R. A. Mendelsohn F. A. Smith A. I. Brennan M. E. Tetaz T. J. (1997). The globin fragment LVV-hemorphin-7 is an endogenous ligand for the AT4 receptor in the brain. J. Neurochem. 68 (6), 2530–2537. 10.1046/j.1471-4159.1997.68062530.x 9166749

[B35] Moisan S. Harvey N. Beaudry G. Forzani P. Burhop K. E. Drapeau G. (1998). Structural requirements and mechanism of the pressor activity of Leu-Val-Val-hemorphin-7, a fragment of hemoglobin beta-chain in rats. Peptides 19 (1), 119–131. 10.1016/S0196-9781(97)00273-8 9437744

[B36] Nyberg F. Sanderson K. Glämsta E. L. (1997). The hemorphins: a new class of opioid peptides derived from the blood protein hemoglobin. Biopolymers 43 (2), 147–156. 10.1002/(SICI)1097-0282(1997)43:2<147::AID-BIP8>3.0.CO;2-V 9216251

[B37] Pin J.-P. Kniazeff J. Liu J. Binet V. Goudet C. Rondard P. (2005). Allosteric functioning of dimeric class C G-protein-coupled receptors. FEBS J. 272 (12), 2947–2955. 10.1111/j.1742-4658.2005.04728.x 15955055

[B38] Piot J. M. Zhao Q. Guillochon D. Ricart G. Thomas D. (1992). Isolation and characterization of two opioid peptides from a bovine hemoglobin peptic hydrolysate. Biochem. Biophys. Res. Commun. 189 (1), 101–110. 10.1016/0006-291X(92)91531-T 1449465

[B39] Sanderson K. Nyberg F. Khalil Z. (1998). Modulation of peripheral inflammation by locally administered hemorphin-7. Inflamm Res. 47 (2), 49–55. 10.1007/s000110050266 9535541

[B40] Szikra J. Benyhe S. Orosz G. Darula Z. Piot J. M. Fruitier I. (2001). Radioligand binding properties of VV-hemorphin 7, an atypical opioid peptide. Biochem. Biophys. Res. Commun. 281 (3), 670–677. 10.1006/bbrc.2001.4397 11237710

[B41] Tang W. Strachan R. T. Lefkowitz R. J. Rockman H. A. (2014). Allosteric modulation of β-arrestin-biased angiotensin II type 1 receptor signaling by membrane stretch. J. Biol. Chem. 289 (41), 28271–28283. 10.1074/jbc.M114.585067 25170081 PMC4192482

[B42] Teixeira L. B. Parreiras-e-Silva L. T. Bruder-Nascimento T. Duarte D. A. Simões S. C. Costa R. M. (2017). Ang-(1-7) is an endogenous β-arrestin-biased agonist of the AT 1 receptor with protective action in cardiac hypertrophy. Sci. Rep. 7 (1), 11903. 10.1038/s41598-017-12074-3 28928410 PMC5605686

[B43] Tóth A. D. Turu G. Hunyady L. Balla A. (2018). Novel mechanisms of G-protein-coupled receptors functions: AT1 angiotensin receptor acts as a signaling hub and focal point of receptor cross-talk. Best Pract. Res. Clin. Endocrinol. Metab. 32 (2), 69–82. 10.1016/j.beem.2018.02.003 29678287

[B44] Wan Q. Okashah N. Inoue A. Nehmé R. Carpenter B. Tate C. G. (2018). Mini G protein probes for active G protein-coupled receptors (GPCRs) in live cells. J. Biol. Chem. 293 (19), 7466–7473. 10.1074/jbc.RA118.001975 29523687 PMC5949987

[B45] Wang J. Hanada K. Gareri C. Rockman H. A. (2018). Mechanoactivation of the angiotensin II type 1 receptor induces β-arrestin-biased signaling through Gαi coupling. J. Cell Biochem. 119 (4), 3586–3597. 10.1002/jcb.26552 29231251 PMC5826900

[B46] Wingler L. M. McMahon C. Staus D. P. Lefkowitz R. J. Kruse A. C. (2019). Distinctive Activation Mechanism for Angiotensin Receptor Revealed by a Synthetic Nanobody. Cell. 176 (3), 479–490.e12. 10.1016/j.cell.2018.12.006 30639100 PMC6367718

[B47] Yukhananov R. Y. Glämsta E.-L. Nyberg F. (1994). Interaction of hemorphins with opioid receptors in the rat vas deferens and guinea-pig ileum. Regul. Pept. 53, S239–S242. 10.1016/0167-0115(94)90329-8

[B48] Zhao Q. Piot J. M. (1997). Investigation of inhibition angiotensin-converting enzyme (ACE) activity and opioid activity of two hemorphins, LVV-hemorphin-5 and VV-hemorphin-5, isolated from a defined peptic hydrolysate of bovine hemoglobin. Neuropeptides 31 (2), 147–153. 10.1016/S0143-4179(97)90084-6 9179868

[B49] Zhao Q. Piot J. M. Sannier F. Guillochon D. (1995). Peptic hemoglobin hydrolysis in an ultrafiltration reactor at pilot plant scale generates opioid peptides. Ann. N.Y. Acad. Sci. 750, 452–458. 10.1111/j.1749-6632.1995.tb19995.x 7785876

